# Supplementary material for: Medical students’ perceptions of their preparedness to care for LGBT patients in Taiwan: Is medical education keeping up with social progress?
Source: PLoS One. 2022 Jul 7;17(7):e0270862. doi: 10.1371/journal.pone.0270862 (PMC9262208; doi:10.1371/journal.pone.0270862)
Supplement: S1 File — (DOCX) [file pone.0270862.s001.docx]

Supplementary Material 1: NVIVO Codebook for focus groups & interviews

| Name/Nodes | Description |
| --- | --- |
| Bias | Bias formed in the process of training |
|  | Associating certain diseases and health risks with LGBT patients |
|  | Reflexive thought/behavior built on past experience or learning rather than being biased (other reasons for making associations) |
|  | Other reasons for forming associations between LGBT identity and diseases |
| Curriculum | Courses that touched upon topics related to sexual and gender minorities |
|  | Role-modeling |
|  | Implicit curriculum, including interpersonal & society/community/environment |
| Perceptions and attitudes | All equal; perceiving everyone the same way (regardless of gender identification or sexual orientation. LGBTs are the same as everyone else (except for their gender identities and sexual orientations and in special circumstances) |
|  | Only ask about patients’ sexual orientation or gender identity only when necessary or relevant for diagnostic purposes |
|  | Wide acceptance & self-perceived openness toward LGBT individuals |
|  | LGBT individuals unwilling to say or share with others in the “out group”; less willing to communicate |
|  | Sensitivity to LGBT-related topics |
|  | Respecting privacy of patients |
|  | Making continuous improvements in caring for LGBT patients |
| Preparation/preparedness | Have “heard” or “seen”, but no actual encounters with LGBT patients; won’t know until actual encounters |
|  | Feel prepared due to previous exposures (direct/indirect) to LGBT individuals in daily life |
|  | Feeling prepared mentally, but lacking skillfulness to care for LGBT patients |
