# Supplementary material for: Medical students’ perceptions of their preparedness to care for LGBT patients in Taiwan: Is medical education keeping up with social progress?
Source: PLoS One. 2022 Jul 7;17(7):e0270862. doi: 10.1371/journal.pone.0270862 (PMC9262208; doi:10.1371/journal.pone.0270862)
Supplement: Attachment — Submitted filename: Response to reviewers_R2.docx [file pone.0270862.s003.docx]

Dear Editor and Reviewers,

Thank you for reviewing our revised manuscript again and for providing further comments. The page and line numbers below are listed according to the “Revised Manuscript with Track Changes” file. We would like to respond to all the comments point by point as follows:

**Comments to the Author**

1. If the authors have adequately addressed your comments raised in a previous round of review and you feel that this manuscript is now acceptable for publication, you may indicate that here to bypass the “Comments to the Author” section, enter your conflict of interest statement in the “Confidential to Editor” section, and submit your "Accept" recommendation.

Reviewer #1: (No Response)

Reviewer #2: All comments have been addressed

**Author’s Response (AR): We thank Reviewers 1 & 2 for reviewing our paper again and for providing additional comments. We will address these comments below.**

2. Is the manuscript technically sound, and do the data support the conclusions?

The manuscript must describe a technically sound piece of scientific research with data that supports the conclusions. Experiments must have been conducted rigorously, with appropriate controls, replication, and sample sizes. The conclusions must be drawn appropriately based on the data presented.

Reviewer #1: No

Reviewer #2: Yes

**AR: Since the two reviewers provided different responses, we are unsure which part(s) Reviewer 1 felt did not meet these criteria. We felt our study was conducted rigorously with appropriate sample size for qualitative study. Our focus groups and interviews were properly conducted, transcribed, and analyzed. Our conclusions were also drawn from the data we had collected and presented.**

3. Has the statistical analysis been performed appropriately and rigorously?

Reviewer #1: No

Reviewer #2: Yes

**AR: Similar to our response above, we are unsure which part(s) of our statistical analysis Reviewer 1 felt was not done appropriately and rigorously. We provided demographics of our participants and added in the number of respondents as well as percentages in Table 3 as Reviewer 1 had suggested. As the rest of our analysis are qualitative, additional quantitative analysis would not be feasible.**

4. Have the authors made all data underlying the findings in their manuscript fully available?

The [PLOS Data policy](http://www.plosone.org/static/policies.action#sharing) requires authors to make all data underlying the findings described in their manuscript fully available without restriction, with rare exception (please refer to the Data Availability Statement in the manuscript PDF file). The data should be provided as part of the manuscript or its supporting information, or deposited to a public repository. For example, in addition to summary statistics, the data points behind means, medians and variance measures should be available. If there are restrictions on publicly sharing data—e.g. participant privacy or use of data from a third party—those must be specified.

Reviewer #1: No

Reviewer #2: Yes

**AR: As our data are qualitative transcriptions and contain quotes provided by student respondents, including the full transcriptions might be problematic. Participants were not informed, nor did they provide approval for publication of their full transcriptions. Our transcriptions could also include information that would make the participants identifiable. Thus, we have made our NVIVO codebook for focus groups and interviews available instead as this provide readers complete information of how we coded our transcriptions.**

5. Is the manuscript presented in an intelligible fashion and written in standard English?

PLOS ONE does not copyedit accepted manuscripts, so the language in submitted articles must be clear, correct, and unambiguous. Any typographical or grammatical errors should be corrected at revision, so please note any specific errors here.

Reviewer #1: No

Reviewer #2: Yes

**AR: Similar to previous questions, we are unsure which part(s) Reviewer 1 thought was/were written in unintelligible fashion or were not written in standard English. We went through the manuscript and to the best of our abilities, made minor revisions to language that might be unclear. Among the four researchers, one is an English native speaker, two Chinese and English bilingual speakers (one received complete education and one received master and PhD degrees in English speaking countries). We also asked one native English- speaking scholar to read through the paper again to help ensure the manuscript is intelligible and written in standard English.**

6. Review Comments to the Author

Please use the space provided to explain your answers to the questions above. You may also include additional comments for the author, including concerns about dual publication, research ethics, or publication ethics. (Please upload your review as an attachment if it exceeds 20,000 characters)

Reviewer #1: Response to my comments (6 and 7) are non-satisfactory.
They need to present clear categories with percentages in table 3 and cross tabulate against the National and the Private schools (using a Fisher or a Chi square test at leas).
The Results, as they are listed in the Abstract are unsupported results if no analysis is done.

**AR: Reviewer 1’s previous comments were regarding the provision of descriptions of the two medical schools involved (comment 6) and results showing percentages for the different categories (comment 7).**

**In our previous response, we confirmed that sampling of focus groups was done randomly after categorization of years of study to ensure that we would at least have one group of students per cohort. However, gender across focus groups was by random. We also added descriptions of the two medical schools involved as follows:**

**On pg. 5-6, lines 100-103 and in the *Note* section of Table 2, we described the compositions of medical programs in Taiwan. The description is applicable to the majority of medical schools in Taiwan, including the two that are included in the study. These descriptions are listed below:**

**“In Taiwan, most medical schools are undergraduate entry level, with years one to four as the pre-medical and pre-clinical stages and years five to six as the clinical stage. All medical graduates attend the compulsory two-year postgraduate (PGY) general medicine training program.” (pg. 5, lines 97-100)**

**“Taiwanese medical schools generally admit high school graduates. Starting from 2013, the original 7-year medical education program changed to 6-years, with the original final internship year moved to postgraduate clinical training (PGY). As the study was conducted in 2015-2017 before the implementation of the two-year PGY program, only the first PGY year was included in the study.” (Table 2)**

**In addition, we added the phrase “one public and one private” and the word “southern” to provide additional descriptions:**

**“…was administered to 1545 medical students from one public and one private medical schools in southern Taiwan…” (pg. 6, lines 102-103)**

**Also, as Taiwan’s medical schools (both private and public) adopt uniform selection criteria, the demographics of students at the two schools are relatively similar.**

**Per the reviewer’s suggestion, we also added the total number of respondents included in discussions of each category in parentheses. To provide a clearer presentation, we have bolded the text for the categories and in addition, added percentages to tabulate the distributions of quotations from public and private schools.**

**However, as the purpose of our study is to explore students’ qualitative responses regarding their perceptions and not to compare quantitatively between the proportion of responses from public and private schools, we feel that including a tabulation would not contribute substantial meaning to help readers understand the results and might detract readers’ attention from the study’s main results. Nevertheless, we added these percentages to Table 3.**

Reviewer #2: Authors have complied all the issues raised earlier

one minor correction they should do
Introduction - Line 80 (Page 15)
The current study is part of a larger project that used mixed methods to examine Taiwan medical students’ perspectives in caring for patients from diverse groups
Comment: is a part of larger project that used mixed method should be delete, it is confusing , better to say ‘qualitative’ method was employed ............

**AR: We changed introduction (pg. 15, line 80) to say the following:**

**“The current study used qualitative method to examine medical students’ preparedness to care for LGBT patients. This study is also part of a larger project that used mixed methods to examine…”**

**In addition to clarifying that the current study employs qualitative method, we felt we should still inform readers that this study originally stemmed from a larger study, which employed mixed methods. Readers can thus refer to our previous study if necessary. Thus, we revised the sentence as shown above.**
